# Simultaneous Alteration of the Circadian Variation of Memory, Hippocampal Synaptic Plasticity, and Metabolism in a Triple Transgenic Mouse Model of Alzheimer’s Disease

**DOI:** 10.3389/fnagi.2022.835885

**Published:** 2022-03-31

**Authors:** António M. Carvalho da Silva, Cristina Lemos, Henrique B. Silva, Ildete L. Ferreira, Angelo R. Tomé, A. Cristina Rego, Rodrigo A. Cunha

**Affiliations:** ^1^CNC-Center for Neuroscience and Cell Biology, University of Coimbra, Coimbra, Portugal; ^2^IIIUC-Institute for Interdisciplinary Research, University of Coimbra, Coimbra, Portugal; ^3^Faculty of Medicine, University of Coimbra, Coimbra, Portugal

**Keywords:** circadian, Alzheimer’s disease, behavior, LTP, mitochondria, Zeitgeber, hippocampus

## Abstract

Alzheimer’s disease (AD) is characterized by progressive memory deficits accompanied by synaptic and metabolic deficits, namely of mitochondrial function. AD patients also display a disrupted circadian pattern. Thus, we now compared memory performance, synaptic plasticity, and mitochondria function in 24-week-old non-transgenic (non-Tg) and triple transgenic male mice modeling AD (3xTg-AD) at Zeitgeber 04 (ZT-4, inactive phase) and ZT-16 (active phase). Using the Morris water maze test to minimize the influence of circadian-associated locomotor activity, we observed a circadian variation in hippocampus-dependent learning performance in non-Tg mice, which was impaired in 3xTg-AD mice. 3xTg-AD mice also displayed a lack of circadian variation of their performance in the reversal spatial learning task. Additionally, the amplitude of hippocampal long-term potentiation also exhibited a circadian profile in non-Tg mice, which was not observed in 3xTg-AD mice. Moreover, cerebral cortical synaptosomes of non-Tg mice also displayed a circadian variation of FCCP-stimulated oxygen consumption as well as in mitochondrial calcium retention that were blunted in 3xTg-AD mice. In sum, this multidimensional study shows that the ability to maintain a circadian oscillation in brain behavior, synaptic plasticity, and synaptic mitochondria function are simultaneously impaired in 3xTg-AD mice, highlighting the effects of circadian misalignment in AD.

## Introduction

Alzheimer’s disease (AD) is characterized by a progressive memory deterioration, involving a loss of synaptic efficiency typified by altered patterns of synaptic plasticity and a hypometabolic profile in afflicted regions, namely in the hippocampus (Scheltens et al., 2016). AD patients also display alterations of their circadian rhythms (Harper et al., 2005), which often predates the onset of cognitive deficits (Musiek et al., 2018) and seems to be a predictor of the evolution into AD (Targa et al., 2021). Indeed, memory performance critically requires a functional circadian rhythm (Ruby et al., 2008; reviewed in Gerstner and Yin, 2010), which are entrained by a system of endogenous molecular clocks defining circadian rhythms of gene expression and metabolic activity that coordinate internal biological processes, synchronizing them with external environmental cycles (Yamazaki et al., 2000; reviewed in Mohawk et al. (2012)).

Animal models were paramount to establish the association between the dysfunction of metabolic processes and of synaptic plasticity with memory function (Walsh and Selkoe, 2004; Small et al., 2011). Animal models of AD also recapitulate alterations of circadian rhythms (reviewed in Sheehan and Musiek, 2020), but they have not been thoughtfully exploited to detail the interplay between alterations of memory, synaptic plasticity, and brain metabolism in AD. The triple transgenic mouse model of AD (3xTg-AD) shows circadian phenotypes more consistent with human AD (Sheehan and Musiek, 2020); they also present an accelerated deterioration of synaptic bioenergetics accompanied by altered mitochondria (Yao et al., 2009; Espino de la Fuente-Muñoz et al., 2020) and we have previously shown that they display parallel alterations of hippocampal synaptic plasticity with the onset and recovery of memory performance (Silva et al., 2018). Thus, we now used male 3xTg-AD mice with early but established memory dysfunction (at 24 weeks of age) to investigate if there were parallel alterations of hippocampal-dependent behavior, hippocampal synaptic plasticity, and mitochondria-associated metabolic alterations in cerebrocortical synaptosomes at two representative times of the day, Zeitgeber (ZT) 04 (4 h after lights on) and ZT 16 (4 h after lights off).

## Materials and Methods

### Animals

Non-Tg (C57BL6/129sv) and 3xTg-AD mice, harboring three cumulative mutations in TauP301L, APPSwe, and γ-secretase (PS1M146V) to model AD (Oddo et al., 2003), were obtained from Dr. Frank LaFerla (Univ. California, Irvine, United States). They were bred and maintained in our animal house as two independent colonies. We selected using male mice since circadian alterations were more consistent and evident in male compared to female 3xTg-AD mice (Wu et al., 2018). They were housed in groups of up to 5 under a 12 h light/12 h dark cycle, a constant temperature of 23–27°C and constant relative humidity, with *ad libitum* access to water and food in an enriched environment. All experiments were carried out at Zeitgeber (ZT) 04 (4 h after lights on, i.e., inactive phase) and ZT16 (4 h after lights off, i.e., active phase) in 24-week-old mice, where we have previously characterized the alterations of hippocampal-dependent memory deficits together with alterations of hippocampal synaptic plasticity (Silva et al., 2018) as well as increases of cerebral cortical oxidative stress (Mota et al., 2015). All animal procedures were approved by the Ethical Committee of the Center for Neurosciences and Cell Biology and *Direção Geral de Veterenária* (ORBEA_243_2020), in accordance with the approved animal welfare guidelines (FELASA) and European legislation (2010/63/EU).

### Behavioral Testing

At the age of 8 weeks, mice were subjected to habituation to handling and experimental user interaction which was performed only in the dark cycle. All animal husbandry procedures and experimental testing were performed under dim red light conditions, to avoid phase shifts in circadian regulation. Animals were divided into active phase (ZT16) and inactive phase group (ZT04), and all experiments were performed in a 2-h time interval from the respective ZT.

The Morris water maze (MWM) test was carried out as previously described (Moreira-de-Sá et al., 2020). In brief, the test consisted of a circular pool filled with water rendered opaque by tempera paint, maintained at room temperature (i.e., 20–21°C) with a fixed platform hidden just below the water surface level and visual cues hung on the walls of the testing room as topographical cues. Each animal was placed into the various quadrants of the pool and we first performed at day 0 a visible platform trial to ensure that all animals possessed the visual accuracy required to perform the task. Each animal then performed four trials per day for four consecutive days; in each trial, the animal was allowed to swim for a period of 60 s until it found the hidden platform; when it reached the platform, the animal was kept for 10 s on the platform. On the 5th day, the platform was removed from the pool, and each animal was allowed to swim for a 60 s period. Several parameters were measured, namely the time spent in the target quadrant, the number of crosses over the platform location, the time elapsed till reach the location of the platform, the mean speed, and the time spent in the quadrant opposite to that having the platform.

We next carried out a reversal spatial learning test (D’Hooge and De Deyn, 2001), which allows assessing the cognitive flexibility necessary to extinguish old memories and form a new memory (Hebb, 1949). The hidden platform was re-located to the opposite quadrant and each animal was allowed to swim for 60 s or until it found the relocated hidden platform. A total of four trials were performed and time elapsed until finding the platform, distance traveled in the different quadrants, and mean swimming speed were recorded. Experiments were recorded and analyzed using the ANY-maze^®^ software (Stoelting, United States).

### Electrophysiological Recordings of Synaptic Plasticity

The extracellular electrophysiological recordings were performed as previously described (Silva et al., 2018) in acute transverse slices from the dorsal hippocampus. For the active phase group (ZT16), slices were prepared at ZT15, and recordings were made between Zeitgeber 16 and 18. For the inactive phase group (ZT04), slices were prepared at ZT03, and recordings were made between Zeitgeber 04 and 06.

After deep anesthesia with an intraperitoneal injection of avertin (250 mg/kg), mice were transcardially perfused with 25–30 mL of room temperature carbogenated N-methyl-D-glucamine—artificial cerebral spinal fluid (NMDG-aCSF) solution (92 mM NMDG, 2.5 mM KCl, 1.25 mM NaH_2_PO_4_, 30 mM NaHCO_3_, 20 mM HEPES, 25 mM glucose, 2 mM thiourea, 5 mM Na-ascorbate, 3 mM Na-pyruvate, 0.5 mM CaCl_2_ and 10 mM MgSO_4_, titrated to pH 7.3–7.4 with concentrated hydrochloric acid 37%). The brain was gently extracted and placed into cold NMDG-aCSF solution for 1 min. Hippocampi were dissected free, in an ice-cold NMDG-aCSF solution and gassed with 95% O_2_ and 5% CO_2_. Slices with 400 μm thickness were obtained with a McIllwain chopper and allowed to recover in a Harvard Apparatus resting chamber filled with gassed (95% O_2_ and 5% CO_2_) aCSF solution (124 mM NaCl, 3 mM KCl, 1.25 mM NaH_2_PO_4_, 10 mM glucose, 26 mM NaHCO_3_, 1 mM MgSO_4_, 2 mM CaCl_2_) for 30 min at 35°C and for 30 min at room temperature.

Individual slices were transferred to a 1 mL capacity-recording chamber for submerged slices and continuously superfused at a rate of 3 mL/min with gassed aCSF kept at 30.5°C. Orthodromically-evoked field excitatory postsynaptic potentials (fEPSP) were recorded through an extracellular microelectrode pipette filled with 4 M NaCl (2–4 MΩ resistance) placed in the *stratum radiatum* of the CA1 area, upon stimulation with a bipolar concentric electrode placed on the Schaffer collaterals, applying rectangular pulses of 0.1 ms every 20 s. No GABAergic inhibitors were present. Responses were collected with an ISO-80 amplifier (World Precision Instruments, Hertfordshire, United Kingdom) and digitized using an ADC-42 board (Pico Technologies, Pelham, NY, United States). Averages of 3 consecutive responses were acquired with the winLTP software (Anderson and Collingridge, 2007) to quantify the initial slope of the averaged fEPSPs. Input/output (I/O) curves were generated by varying the stimulus intensity from 0 to 100 μA in 10 μA steps, allowing to select a stimulation intensity to evoke an fEPSP of about 40% of maximal amplitude. After obtaining a stable basal line for 10 min, long-term potentiation (LTP) was evoked by a high-frequency stimulation (HFS) train of 100 Hz and 1 s duration. LTP was quantified as the percentage change between the fEPSP slopes 60 min after and 15 min before the train.

### Preparation of Cerebrocortical Synaptosomes

Synaptosomes, a preparation most suitable for studying synaptic function (Raiteri and Raiteri, 2000), were obtained as previously described (Silva et al., 2018) from cerebrocortical tissue collected at ZT04 and ZT16 from 3xTg-AD and non-Tg mice. In brief, cerebral cortical tissue was placed into centrifuge tubes filled with 5 mL ice-cold 0.32 M sucrose solution containing 1 mM EDTA, 10 mM HEPES, 1 mg/mL bovine serum albumin (BSA), at pH 7.4 and homogenized using a 15-mL Teflon-glass tissue grinder. The mixture was centrifuged at 3,000 × *g* for 10 min at 4°C, the supernatant was collected and centrifuged again at 14,000 × *g* for 12 min at 4°C. The pellet was then resuspended in 1 mL of a 45% (v/v) Percoll in saline solution (156 mM NaCl, 3 mM KCl, 2 mM MgSO_4_, 1.25 mM KH_2_PO_4_, 2 mM CaCl_2_, 10 mM glucose, and 10 mM HEPES, pH adjusted to 7.35) and centrifuged at 14,000 × *g* for 2 min at 4°C. The pellet was washed twice with saline solution and kept at 0–4°C.

#### Oxygen Consumption

Oxygen consumption was measured using a Clark-type electrode (YSI Model 5331, Yellow Springs Inst.) in a closed glass chamber equipped with magnetic stirring and maintained at 30°C, as previously described (Santos et al., 2000). The experiments were carried out in the cerebral cortex since hippocampal tissues were used for electrophysiological analysis, thus enabling to carry out all analyses in the same animals. Basal oxygen consumption was evaluated in cerebral cortical synaptosomes collected either at ZT04 or ZT16 from non-Tg or 3xTg-AD mice (∼0.4 mg/mL) in 1 mL of saline solution. The mitochondrial uncoupler cyanide p-(trifluoromethoxy) phenylhydrazone (FCCP, 1 μM; Sigma) was added, and oxygen consumption was recorded for an additional 2 min. Oxygen consumption was calculated assuming an oxygen concentration of 230 nmol O_2_/mL.

#### Mitochondrial Membrane Potential (Δψm)

Mitochondrial membrane potential in synaptosomes was measured as previously described (Amorim et al., 2017), using 10 μg of synaptosomal protein, determined using the bicinchoninic acid (BCA) protein assay kit from Biorad. Briefly, cortical synaptosomes were incubated with the fluorophore tetramethylrhodamine methyl ester (TMRM^+^, 300 nM; from Invitrogen) for 30 min at 30°C, followed by a spin down at 7,500 × *g* for 1 min at room temperature and resuspension in saline solution. Synaptosomes were plated on a 96-well plate, and fluorescence was read at 573 nm upon excitation at 548 nm. We first established a 5 min baseline, before triggering the depolarization of mitochondria by addition of FCCP (1 μM) and of the ATP synthase inhibitor oligomycin (1 μg/mL; Sigma), which effects were calculated after 3 min.

#### Mitochondrial Calcium Determination

MitoCa^2+^ retention can be indirectly measured by the rise in Fura-2 fluorescence after complete mitochondrial depolarization (and subsequent release of mitoCa^2+^ into cytosol) observed following exposure to FCCP stimulation in the presence of oligomycin. After a washing step with saline medium, cortical synaptosomes were loaded with 5 μM of the ratiometric probe Fura-2/AM in the same medium for 30 min at 30°C, followed by a spin down at 7,500 × *g* for 1 min at room temperature. Synaptosomes (10 μg) were plated on a 96-well plate and Fura-2 fluorescence measured using a Spectrofluorometer Gemini EM (Molecular Devices) microplate reader at 340/380 nm excitation and 510 nm emission wavelengths under basal condition and after stimulation with 1 μM FCCP and 1 μg/mL oligomycin. All plotted values were normalized for the initial baseline value.

### Statistics

Data are mean ± SEM values or mean (95% confidence interval) of the number of mice included in each experiment (n). Data with one condition and one variable were analyzed with a Student’s *t*-test. Data with more than one variable (genotype and treatment) were analyzed with a two-way ANOVA followed by Newman–Keuls *post hoc* test. Unless otherwise indicated the significance level was 95%.

## Results

Since memory performance, hippocampal synaptic plasticity, and cortical mitochondria dysfunction are three critically affected processes at the onset of AD, we assessed the circadian variation of these three cardinal parameters in non-Tg and 3xTg-AD mice at two different times of the day, namely Zeitgeber (ZT) 04 (inactive period) and 16 h (active period).

### Circadian Variation of Learning and Memory Deficit

**FIGURE 1:**
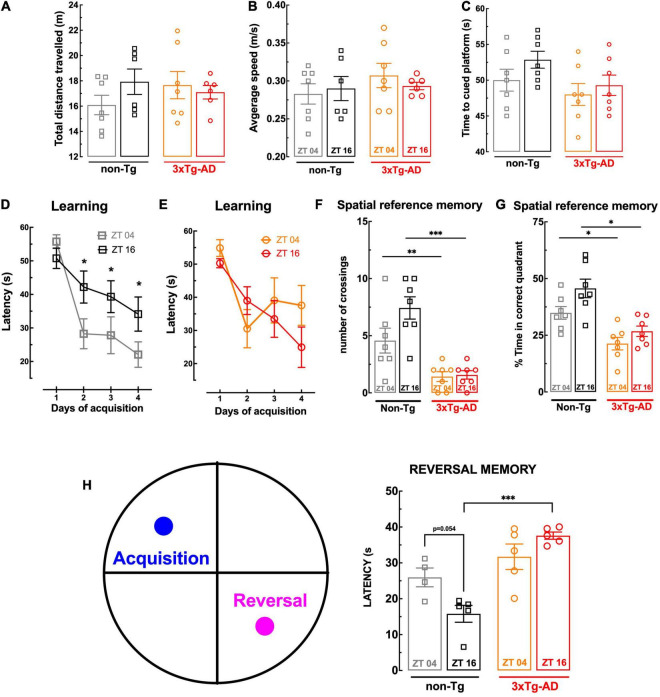
Behavior comparison of non-Tg and 3xTg-AD mice at ZT04 (inactive phase) and ZT16 (active phase). There was no effect of genotype or circadian period on swimming activity, assessed as the total distance traveled **(A)** and of the average speed **(B)** in the Morris water maze (MWM), or visual capabilities, assessed as the latency to reach the visibly tagged platform in the MWM **(C)**. The 3xTg-AD mice presented a poor learning performance in the MWM than non-Tg mice at ZT04 but not at ZT16 **(D,E)**, and there was a circadian variation of learning in non-Tg, which was not present in 3xTg-AD **(D,E)**. Spatial reference memory assessed in the MWM was poorer in 3xTg-AD than non-Tg mice at both times of the day without circadian variations **(F,G)**, whereas reversal memory in the MWM displayed a circadian variation in non-Tg mice, which was not present in 3xTg-AD mice, which displayed a poorer performance at ZT16 but not at ZT04 **(H)**. Data are mean ± SEM of 4–7 mice per group, as indicated by the individual symbols (non-Tg at ZT04 in gray squares, non-Tg at ZT16 in black squares, 3xTg-AD at ZT04 in orange circles, and 3xTg-AD at ZT16 in red circles). Statistical analysis: *p < 0.05, ^**^p < 0.01; ^***^p < 0.001 vs. other ZT or between indicated bars using a Newman–Keuls *post hoc* test after a two-way ANOVA.

We first compared the learning and memory performance of non-Tg and 3xTg-AD mice using the Morris water maze test, to limit the possible influence of the known circadian variation of general activity and locomotion (Valentinuzzi et al., 2000; Albrecht and Oster, 2001; Kopp, 2001), which is less evident in a mildly aversive condition of water exposure to mice. As expected, there was no major variation between non-Tg and 3xTg-AD mice at both ZT of the total swam distance [genotype: *F*_(1,22)_ = 0.174, *p* = 0.681; ZT: *F*_(1,22)_ = 0.519, *p* = 0.479; Figure 1A], of the swimming speed [genotype: *F*_(1,22)_ = 1.01, *p* = 0.326; ZT: *F*_(1,22)_ = 0.0599, *p* = 0.809; Figure 1B] and of the latency to reach the visibly tagged platform [genotype: *F*_(1,22)_ = 2.13, *p* = 0.159; ZT: *F*_(1,22)_ = 3.83, *p* = 0.063; Figure 1C], indicating that both groups have similar motor and visual capabilities in the tested conditions.

In accordance with our previous findings (Silva et al., 2018) and of others using the Morris water maze test (Billings et al., 2005), 3xTg-AD mice with 24 weeks of age tended to display learning deficits, as suggested by a tendency for a slower learning when compared to non-Tg mice (Figures 1D,E). As shown in Figure 1D, non-Tg animals exhibited a circadian profile in spatial learning, with a significantly lower escape latency time to reach the hidden platform at ZT04 than at ZT16 (*p* = 0.047). Notably, the analysis of 3xTg-AD mice learning curves showed no difference between the two Zeitgebers (*p* = 0.877; Figure 1E).

When testing the retention of spatial memory upon removal of the hidden platform, non-Tg mice performed significantly better than 3xTg-AD mice, independently of the ZT (Figures 1F,G). For instance, at ZT04, the number of crossings in the quadrant previously containing the platform of non-Tg mice (4.57 ± 1.09, *n* = 7) was larger (*p* = 0.028) than for 3xTg-AD mice (1.43 ± 0.429, *n* = 7) (Figure 1F); likewise, the percentage of time spent in the “correct” quadrant by non-Tg mice (34.9 ± 2.79%, *n* = 7) was larger (*p* = 0.005) than for 3xTg-AD mice (21.3 ± 2.77%, *n* = 7) (Figure 1G). There is a tendency for a circadian variation in the ability of non-Tg mice to cross more frequently (*p* = 0.074) and spend more time (*p* = 0.053) in the correct quadrant at ZT16 than at ZT04, which was not evident for 3xTg-AD mice (*p* = 0.805 for the number of crossings between and *p* = 0.165 for the percentage time in the correct quadrant between ZT04 and ZT16).

In order to probe cognitive flexibility, we performed a reversal-learning extension of the Morris water maze test, by relocating on day 6 the platform to the opposite quadrant. As shown in Figure 1H, non-Tg mice displayed a clear tendency for a circadian variation of the performance in this reversal spatial learning test (*p* = 0.054) that was not present in 3xTg-AD mice (*p* = 0.149), which had a statistically significant decline of cognitive flexibility compared to non-Tg mice only at ZT16 (Figure 1H).

### Circadian Variation of Hippocampal Synaptic Plasticity

**FIGURE 2:**
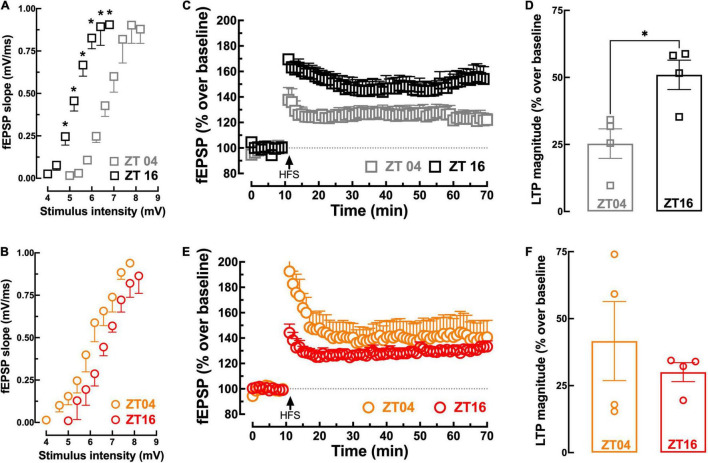
Comparison of synaptic transmission and plasticity in hippocampal slices from non-Tg and 3xTg-AD mice collected near ZT04 (inactive phase) and ZT16 (active phase). The input/output curves presenting the field excitatory post-synaptic responses (fEPSP, measured as their slope recorded in the dendritic field of CA1 pyramidal neurons), with increasing stimulation intensity of the afferent Schaffer fibers, show that there was a circadian difference of synaptic transmission with a greater synaptic efficiency at ZT16 than at ZT04 in non-Tg mice **(A)**, which was not present in 3xTg-AD mice **(B)**. The time course of variation of fEPSP responses (expressed as percentage variation of the average basal values) **(C)** shows that a high-frequency stimulation (HFS, 100 Hz for 1 s) train triggered a persistent increase of the fEPSP response (long-term potentiation, LTP) with a magnitude larger at ZT16 than at ZT04 in slices from non-Tg mice **(C)**, which average values are displayed in the bar graph **(D)**. This circadian variation was not present in 3xTg-AD mice, as illustrated in the time course **(E)** and corresponding average values of LTP magnitude **(F)**. Data are mean ± SEM of 4–5 mice per group, as indicated by the individual symbols (non-Tg at ZT04 in gray squares, non-Tg at ZT16 in black squares, 3xTg-AD at ZT04 in orange circles, and 3xTg-AD at ZT16 in red circles). Statistical analysis: *p < 0.05 vs. other ZT or between indicated bars using a Student’s *t*-test.

We next compared in non-Tg and 3xTg-AD mice the pattern of circadian variation of hippocampal long-term potentiation (LTP), which is a purported neurophysiological trait of memory processing (see Martin et al., 2000; Lynch, 2004). We first assessed if the basal excitatory synaptic transmission in Schaffer fibers-CA1 synapses of hippocampal slices displayed a circadian variation. As shown in Figure 2A, input–output (I-O) curves showed an increased (*p* = 0.025; comparing EC_50_ values, *n* = 3–4) synaptic strength at ZT16 compared to ZT04 in non-Tg mice; in contrast, in 3xTg-AD mice, the input–output (I-O) were nearly superimposable at the two tested ZT (*p* = 0.965; comparing EC_50_ values, *n* = 4; Figure 2B). The high-frequency stimulation (HFS)-induced LTP in hippocampal slices revealed a similar alteration of the impact of circadian time. Thus, in non-Tg mice, the magnitude of LTP in hippocampal slices collected at ZT16 (51.0 ± 5.47%, *n* = 4) was larger (*p* = 0.016) than at ZT04 (25.3 ± 5.51%, *n* = 4) (Figures 2C,D). This circadian-related variation was no longer observed in 3xTg-AD mice: here, the magnitude of LTP in hippocampal slices collected at ZT04 (42.1 ± 15.1%, *n* = 4) was not significantly different (*p* = 0.487) from LTP magnitude at ZT16 (30.0 ± 3.54%, *n* = 4) (Figures 2E,F). Furthermore, as expected based on our previous work, LTP magnitude was lower (*p* = 0.018) in 3xTg-AD mice compared to non-Tg mice at ZT16 (Figures 2C–F). Although these electrophysiological alterations in the hippocampus of this AD mouse model are compatible with the predominant dysfunction of glutamatergic synapses in early AD (Kirvell et al., 2006; Canas et al., 2014), the lack of GABAergic inhibitors does not exclude a contribution of an altered inhibitory transmission (reviewed in Palop and Mucke (2016)).

### Circadian Variation of Mitochondrial Function in Cortical Synapses

Circadian rhythms impact brain metabolism, namely mitochondrial oxygen consumption (Simon et al., 2003), in general agreement with the evidence that mitochondrial respiration is entrained by circadian rhythms in different tissues (reviewed in de Goede et al., 2018). Different synaptic functions also display a circadian variation (e.g., Barnes et al., 1977; Liston et al., 2013; Jasinska et al., 2015; McCauley et al., 2020) as best heralded by the circadian fluctuation of LTP magnitude described above, but it has not been defined if the activity of synaptic mitochondria also varies through the day.

**FIGURE 3:**
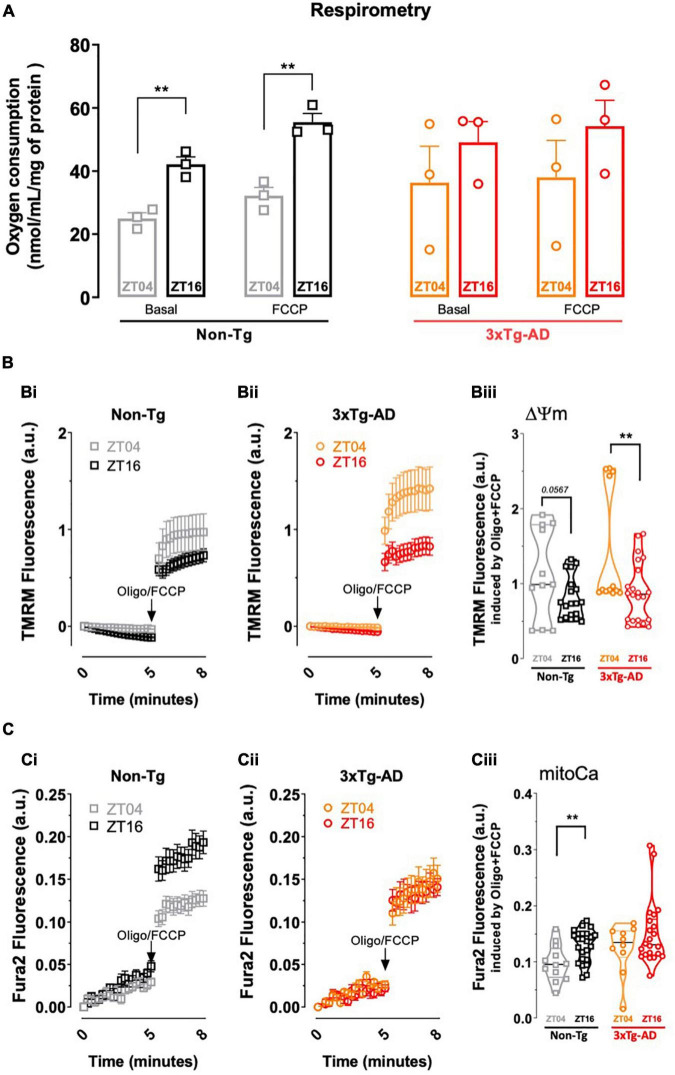
Comparison of mitochondrial function in cortical synapses from non-Tg and 3xTg-AD mice collected near ZT04 (inactive phase) and ZT16 (active phase). **(A)** Oxygen consumption rates (OCR) in cortical synaptosomes isolated from 3xTg-AD vs. non-Tg mice, at ZT04 and ZT16, where the first set of bars represents basal and FCCP induced-OCR at the two ZT’s for non-Tg mice, and the second set of bars represents the same parameters for 3xTg-AD animals. Non-Tg animals display a circadian variation of both basal and FCCP-induced OCR, which is absent in 3xTg-AD. **(B)** Fluorometric analysis of mitochondrial membrane potential in cortical synaptosomes from non-Tg (Bi) and 3xTg-AD mice (Bii) at ZT04 and ZT16, depicted by the representative trace of TMRM^+^ fluorescence. Quantification of TMRM^+^ fluorescence after complete mitochondrial depolarization achieved by addition oligomycin plus FCCP shows a circadian variation for 3xTg-AD animals and a tendency in non-Tg mouse synaptosomes (Biii). **(C)** Fluorimetric analysis of mitoCa retention in cortical synaptosomes from non-Tg (Ci) and 3xTg-AD (Cii) mice at ZT04 and ZT16, depicted by the representative traces of Fura2 fluorescence. Quantification of Fura2 fluorescence following addition of oligomycin plus FCCP shows a circadian variation in non-Tg that is not observed in 3xTg-AD mouse synaptosomal mitochondria (Ciii). Data are presented as the mean ± SEM, *n* = 3–4 mice, each run in triplicates. Statistical analysis: **p < 0.01 vs. other ZT, using a Student’s *t*-test.

As shown in Figure 3A, we now report that oxygen consumption, expressed as nmol/mL/mg of protein, of mitochondria purified from cerebrocortical synapses of non-Tg mice display a larger basal (42.14 ± 2.34, *n* = 3, *p* = 0.0042) and FCCP-stimulated oxygen consumption (55.48 ± 2.73, *n* = 3, *p* = 0.0006) at ZT16 than the basal (23.55 ± 1.8, *n* = 3) and FCCP-stimulated oxygen consumption (32.12 ± 4.52, *n* = 3) at ZT04. Notably, this circadian variation of oxygen consumption is no longer observed in mitochondria purified from cerebrocortical synapses of 3xTg-AD mice (basal oxygen consumption at ZT04 of 36.30 ± 19.89, *n* = 3 and of 49.07 ± 6.58, *n* = 3 at ZT16, *p* = 0.470; FCCP-stimulated oxygen consumption at ZT04 of 37.97 ± 20.09, *n* = 3 and of 54.21 ± 8.19, *n* = 3 at ZT16, *p* = 0.417). Mitochondrial membrane potential, assessed with the potential-sensitive dye TMRM^+^, as previously described (Amorim et al., 2017), displayed a tendency (*p* = 0.057) for circadian variation in purified cerebrocortical synaptosomes of non-Tg mice (Figures 3Bi,iii). TMRM^+^ fluorescence was significantly decreased in 3xTg-AD mice at ZT16 (0.88 ± 0.09, *n* = 3; *p* = 0.0098) compared with ZT04 (1.44 ± 0.23, *n* = 3) (Figures 3Bii,iii), suggesting the presence of uncoupled mitochondria in cortical synapses during the active phase in this AD model. In addition, the increase in mitoCa accumulation in synaptosomes from non-Tg mice at ZT16 (0.13 ± 0.006, *n* = 3; *p* = 0.0057), when compared to ZT04 (0.10 ± 0.009, *n* = 3) (Figures 3Ci,iii), was completely abolished in 3xTg-AD mice (*p* = 0.2094) (Figures 3Cii,iii). Overall, circadian variations in synaptic mitochondrial activity and organelle Ca^2+^ retention are highly affected in 3xTg-AD mice.

## Discussion

The exploitation in the present study of 3xTg mice as a model of AD (Oddo et al., 2003), namely of early AD when used at 6 months of age (Silva et al., 2018), allowed concluding that there is a simultaneous disappearance of the circadian variations of different key features that are deteriorated in early AD, namely memory performance, hippocampal synaptic plasticity and mitochondria function in cortical synapses. This re-enforces the hypothesis that mechanisms defining circadian rhythms may be key determinants of the proficiency of the processes that characterize early AD (Sheehan and Musiek, 2020). Such a hypothesis strengthens the proposal that targeting these processes controlling circadian rhythms may become a particularly powerful overarching strategy to mitigate dysfunctions of afflicted brain regions in early AD.

The main novelty of this work is to integrate into the same “context” the impact of AD and of circadian rhythms on different endpoints that have bilaterally been related but are still not yet integrated on a unified framework. Thus, there is evidence for bilateral relations between mitochondria and LTP (Li et al., 2004, 2010; D’Amelio et al., 2011; Todorova and Blokland, 2017) and of their parallel modification in disease conditions (Camandola and Mattson, 2017; Olesen et al., 2020; Cleland et al., 2021), under the premise that the recruitment of intense neuronal activity involves an intense metabolic budget (Laughlin et al., 1998; Harris et al., 2012) and may be limited by the availability of energy resources (Attwell and Laughlin, 2001). Likewise, there is a tight perceived association between LTP and memory, based on the assumption that the former is the neurophysiological basis of the latter (Martin et al., 2000; Lynch, 2004). Finally, it has seldom been proposed that mitochondria-related metabolic support is a determinant of memory performance (Stranahan and Mattson, 2011). However, there is still no robust evidence to allow establishing a firm causal link between the dysfunction of synaptic mitochondria, synaptic plasticity, and memory. In parallel, there is also good evidence that AD affects each of the three endpoints evaluated in the present study, namely, memory performance, hippocampal synaptic plasticity, and mitochondria function (e.g., Du et al., 2010; Calkins et al., 2011; D’Amelio et al., 2011; Lee et al., 2012), as now confirmed using the 3xTg-AD mice modeling early AD. Finally, there is also compelling evidence showing that alterations of the circadian rhythm can affect metabolism, in particular mitochondria function (reviewed in de Goede et al., 2018), synaptic plasticity (e.g., Harris and Teyler, 1983; Raghavan et al., 1999; McCauley et al., 2020), and behavior function, namely, memory performance (Schmidt et al., 2007; Gerstner and Yin, 2010). However, there is still a lack of a common framework to relate alterations of synaptic mitochondria, synaptic plasticity, and memory performance with alterations of the circadian rhythm in the context of AD.

Previous studies have described alterations of the circadian rhythm associated with AD (Harper et al., 2005; Sheehan and Musiek, 2020; Uddin et al., 2020; Toljan and Homolak, 2021), namely, at the onset of AD (Musiek et al., 2018), and it has even been proposed that circadian variations may be predictors of AD (Targa et al., 2021). In particular, it has been previously reported that AD mouse models display parallel modifications of memory and synaptic plasticity that are associated with a modified circadian rhythm (Lanté et al., 2015; He et al., 2020). Also, although there are previous reports of circadian alterations of oxidative stress associated with memory impairment in AD mouse models (Baño Otalora et al., 2012), and of a circadian oscillation of properties of whole-brain mitochondria (Simon et al., 2003), it has not previously been investigated if there is a circadian variation of the functioning specifically of synaptic mitochondria in early AD. This question is of particular importance since synaptic mitochondria have properties different from non-synaptic mitochondria (Lai et al., 1977; Battino et al., 2000; Brown et al., 2006; Lores-Arnaiz et al., 2016) and they are critical to sustain the functioning and viability of synapses (Du et al., 2010; D’Amelio et al., 2011; Olesen et al., 2020), which undergo a failure at the onset of AD (Scheff et al., 2006; Mecca et al., 2020), to such an extent that AD is considered a synaptic pathology at its onset (Selkoe, 2002). The present findings not only provide the first direct demonstration that synaptic mitochondria display a circadian variation of fundamental metabolic features such as their membrane potential and oxygen consumption, but also show that this circadian variation displayed by synaptic mitochondria is lost in 3xTg-mice modeling early AD. Moreover, our data indicate a simultaneous loss of circadian variation of key properties of synaptic mitochondria in early AD, together with a similar loss of the circadian variation of memory performance and LTP magnitude in 3xTg mice. This provides the first demonstration in the 3xTg mouse model of AD, which shows circadian phenotypes more consistent with human AD (Sheehan and Musiek, 2020), of circadian variations of memory and synaptic plasticity that were shown in other AD rodent models (Baño Otalora et al., 2012; Duncan et al., 2012; Lanté et al., 2015; Stevanovic et al., 2017; Petrasek et al., 2018; He et al., 2020; Fusilier et al., 2021) and in Drosophila AD models (Chen et al., 2014; Long et al., 2014) and only inferred from proteomic analysis in 3xTg-AD mice (Bellanti et al., 2017).

## Conclusion

In conclusion, the present study illustrates a simultaneous impact of an altered circadian rhythm in different variables that are representative of early AD: thus, the present study provides initial findings supporting the integration in the same framework of AD-related alterations of synaptic mitochondria, synaptic plasticity, and memory performance, all under simultaneous control of the circadian rhythm. Although still tentative, this proposal is consistent with the parallel disruption of circadian variations of synaptic mitochondria, synaptic plasticity, and memory performance in this animal model of early AD. This prompts the interest in identifying the mechanisms by which the processes responsible for defining circadian rhythms might simultaneously control the different modifications assessed in this study and certainly others that were not yet investigated. Indeed, although clock genes seem to be able to control mitochondria function (de Goede et al., 2018) as well as synaptic plasticity and memory (Eckel-Mahan and Storm, 2009), it remains to be established if the presently observed loss of circadian variation of synaptic properties and memory is either due to a direct effect of clock genes that are altered in AD (Sheehan and Musiek, 2020) or instead to altered wake-sleep cycles also present in AD (Colwell, 2021) that can affect mitochondria (Smith and Musiek, 2020) as well as synaptic plasticity and memory (Walker and Stickgold, 2006).

## Data Availability Statement

The original contributions presented in the study are included in the article/supplementary material, further inquiries can be directed to the corresponding author/s.

## Ethics Statement

The animal study was reviewed and approved by the Ethical Committee of the Center for Neurosciences and Cell Biology and Direção Geral de Veterenária (ORBEA_243_2020).

## Author Contributions

AC, AR, and RC designed the work and wrote the manuscript, which was reviewed by all authors. CL and AC performed behavioral experiments. AC and HS carried out the electrophysiological recordings. AC and IF carried out the experiments with mitochondria. AC, CL, HS, IF, and AT analyzed the data.

## Conflict of Interest

The authors declare that the research was conducted in the absence of any commercial or financial relationships that could be construed as a potential conflict of interest.

## Publisher’s Note

All claims expressed in this article are solely those of the authors and do not necessarily represent those of their affiliated organizations, or those of the publisher, the editors and the reviewers. Any product that may be evaluated in this article, or claim that may be made by its manufacturer, is not guaranteed or endorsed by the publisher.
